# Leaving No Man Behind: How Differentiated Service Delivery Models Increase Men’s Engagement in HIV Care

**DOI:** 10.34172/ijhpm.2020.32

**Published:** 2020-03-07

**Authors:** Ferdinand C. Mukumbang

**Affiliations:** ^1^South African Medical Research Council, Cape Town, South Africa.; ^2^School of Public Health, University of the Western Cape, Cape Town, South Africa.

**Keywords:** Critical Realism, Differentiated Care Models, Masculinity, HIV Services, South Africa

## Abstract

**Background:** Men demonstrate disproportionately poor uptake and engagement in HIV services with strong evidence linking men’s disinclination to engage in HIV services to their masculinity, necessitating adaptive programming to accommodate HIV-positive men. Differentiated service delivery models (DSDMs) – streamlined patient-centred antiretroviral treatment (ART) delivery services – have demonstrated the potential to improve men’s engagement in HIV services. However, it is unclear how and why these models contribute to men’s reframing of ART-friendly masculinities – a set of attributes, behaviours and roles associated with boys and men that favour the uptake and use of ART. We sought to unveil how and why DSDMs support the formation of ART-friendly masculinities to enhance men’s participation in HIV-related services.

**Methods:** A theory-driven qualitative approach underpinned by critical realism was conducted with 30 adult men using 3 types of DSDMs: facility-based adherence clubs (FACs), community-based adherence clubs (CACs) and quick pharmacy pick-ups (QPUPs). Focus group discussions (FGDs) (6) and in-depth interviews (IDIs) (20) were used to elicit information from purposively selected participants based on their potential contribution to the theory development – theoretical sampling. Recordings were transcribed verbatim in isiXhosa, then translated to English and analysed thematically. Theoretical constructs (themes) related to programme context and generative mechanisms were distilled and linked by retroduction and abductive thinking to formulate explanatory theories.

**Results:** Three bundles of mechanisms driving the adoption of ART-friendly masculinities by men using DSDMs were identified. (1) DSDMs instil a* sense of cohesion* (social support and feeling of connectedness), which enhances their reputational masculinity – having the know-how and being knowledgeable. (2) DSDMs provide a *sense of assurance* by providing reliable, convenient, stigma-free services, which makes men feel strong and resilient (respectability identity). (3) Through *perceived usefulness* , the extent to which an individual believes the model enhances their disease management, DSDMs enhance men’s ability to be economically productive and take care of their family (responsibility identity).

**Conclusion:** DSDMs enhance the refashioning of ART-friendly versions of masculinity, thus improving men’s engagement in HIV services. Their effectiveness in refashioning men’s masculinities to ART friendly masculinities can be improved by ensuring conducive conditions for group interactions and including gender-transformative education to their existing modalities.

## Introduction

Men demonstrate disproportionately poor uptake and participation in HIV services, constituting ‘a blind spot’ in the fight against HIV and AIDS.^1^ Men are more unlikely to take part in testing services, initiate antiretroviral treatment (ART) with more advanced HIV disease, show worse retention in care and adherence to treatment behaviours and, consequently, have worse health outcomes compared to women.^2-5^ These differences in experiencing HIV services appear to relate more to gender norms than to health system factors.^6^ Therefore, apart from the structural barriers to engagement^ [1]^ in HIV services such as distance to the facility, inconvenient hours, sigma, poverty and perceptions that facilities provide women-centred services,^8^ there is strong evidence linking men’s disinclination to engage in HIV care to masculinity.^3,6,9,10^

‘Masculinity is the set of local beliefs and practices that capture what it means in a particular context to be a man.’^3^ Three versions of masculinity have been described (1) responsibility – taking care of one’s family, economic productivity; (2) respectability – being strong, resilient, disease-free; and (3) reputational – highly sexual, be and act in control and having the know-how.^10,11^ Within each society, there exists varied idealised types of masculinity known as hegemonic masculinity. According to Colvin,^3^ most men in that setting implicitly strive to achieve this level of masculinity, which justifies men’s dominance within a broader patriarchal social order. Men’s enactment of the different versions of masculinity has subordinating roles, with male authority limiting their ability to show vulnerability.^12^ These unhealthy constructions of masculinities and patriarchies stifle healthcare access even in times of vulnerability and ill-health.^11,12^ Therefore, behaviours that undermine men’s health are ‘signifiers of masculinity.’^13^

The view of men always being strong and healthy constitutes challenges for men when faced with HIV-positive status.^14^ According to Russell,^15^ ‘HIV and its treatment can undermine masculine identities and consequently men are frequently less able to admit there is a problem, seek support or remain engaged with treatment’ (p. 1199). Loss of their authority, weakened provider role and reliance on others lead to a “dented” masculinity.^4,16^ Nevertheless, under conducive conditions and provided with opportunities, ‘men can renegotiate and critically engage with social representations of what constitutes a “real man” in a particular context.’^11^ This means that men’s concerns about masculinity can be harnessed to encourage healthy “masculine” behaviour.^17^ ART interventions offer men a more reflexive approach to reconstruct masculinities as their physical recovery increase their attentiveness to health.^18^ Therefore, as men adjust to and better manage their condition, they feel comfortable and reformulate ART friendly masculine identities^15^ – a set of attributes, behaviours and roles associated with boys and men that favour the uptake and use of ART.

In South Africa, gender-transformative interventions such as “One Man Can,” a rights-based gender equality and health programme intervention, has shown success in reducing masculinity-related barriers to engaging in HIV services.^19^ While the solutions to enhancing men’s participation in HIV services lie principally at the social level, the health system could play its part in providing HIV services that enable earlier and easier use by men.^6,20^ To this end, community-based outreach programmes and responsive male-friendly health services have been considered in South Africa and other sub-Saharan countries.^3^ For example, the “Male After Hours Service” programme at Médecins Sans Frontières’ Ubuntu ART Clinic in the Western Cape province of South Africa, which provides after-hour services only to men and run by male healthcare workers. Most prevalent, however, are responsive interventions such as differentiated service delivery models (DSDMs) – streamlined patient-centred antiretroviral delivery services offering flexible hours, multiple follow-up visits, and convenient access to care.^21^

While various studies conducted in sub-Saharan countries have demonstrated that DSDMs can improve the overall retention in care and adherence to medication for the general population,^22-26^ a recent study revealed that these models have the potential to increase the retention care and adherence to medication among men in particular.^27^ These findings suggest that DSDMs can play an important role in supporting men’s engagement in HIV treatment and care services.^8^ Nevertheless, it is unclear whether, and if so, how DSDMs support men to reformulate ART-friendly versions of masculinity. To this end, the study was designed to unveil a theory explicating how and why DSDMs facilitate men’s refashioning of health-enabling masculinities thus enhancing their engagement in HIV services.

### Differentiated Service Delivery Models

**Figure 1:**
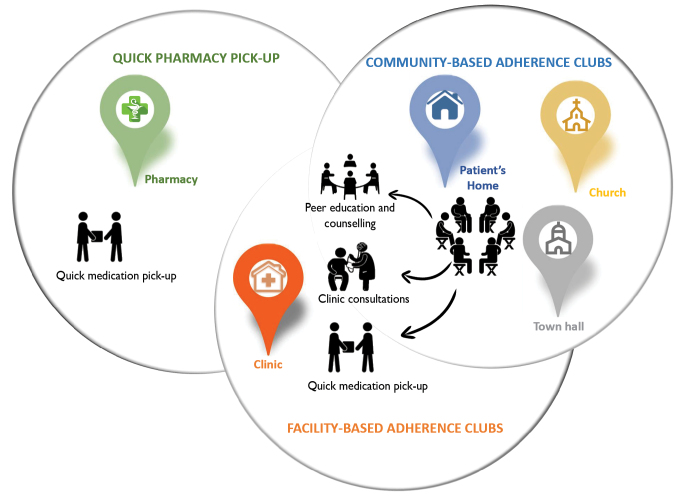
An illustration of the Relationship Between Differentiated ART Delivery Models. Abbreviation: ART, antiretroviral treatment.

‘Differentiated care is a client-centred approach that simplifies and adapts HIV services across the cascade to reflect the preferences and expectations of various groups of people living with HIV (PLHIV) while reducing unnecessary burdens on the health system.’^21^ DSDMs aim to delink clinical visits from ART medication refill visits by decreasing clinical visits to once in 6 months and pharmacy pick-up for HIV medication to once every 2 months or more for stable ART patients.^28^ Stable patients are patients aged 18 years or more, on the same ART regimen for at least 6–12 months, with the 2 most recent consecutive viral loads of the patient undetectable, and having no medical condition requiring regular clinical consultations more than once a year.^29^ DSDMs are ancillary to the mainstream ART care delivery schemes, and they streamline ART service delivery by adapting the care components to the needs of the targeted group.^30^ Three DSDMs are common in South Africa: facility-based adherence clubs (FACs), community-based adherence clubs (CACs) and quick pharmacy pick-up (QPUP). The relationship between these models is illustrated in Figure 1.

### Adherence Club Models

The adherence club model was designed to decongest the healthcare facilities of PLHIV on ART and to encourage peer support among patients. The adherence club model groups stable patients (15–30) who meet and get antiretroviral supply every 2 months.^31^ The groups are facilitated by a lay health worker who provides counselling and education sessions where patients discuss various issues for social-support, weighs the patients and give them pre-packed medication.^32^

The adherence club model can be hosted in a facility (facility-based) and out of the facility (community-based) depending on the availability of space and resources. The model can be implemented in the facility in any safe and secure space where up to 30 patients can be accommodated. Community spaces such as community halls, church halls, libraries and patients’ homes are commonly used to host clubs.^33^ Patients in community clubs have to return to the facility to have their viral load tests and clinical consultation (once a year).

### Quick Pharmacy Pick-Up

QPUP is a fast-lane model of care where patients pick their medication in the facility without a visit to a clinician at each healthcare visit. This model was designed to include more patients and can accommodate 50 patients in 1 QPUP group. The QPUP model is also considered a flexible approach because it allows patients to send people they trust to pick up their medication supply. The client or client-appointed representative goes directly to the pharmacy (open twice weekly) on their scheduled appointment date any time between 4:30 and 7:30 pm.

While FACs, CACs, and QPUP are common DSDMs in South Africa, other models have been designed to suit the needs of other contexts. For example, in Mozambique, community ART groups have been reported to be successful. Community ART groups are self-formed groups of 4 to 8 stable ART patients from the same community area with a group leader who acts as the group reference person responsible for the organisation and information exchange between members. Group members rotate the responsibility of going to the clinic to collect ARV refills for all members of the group thus reducing the patient frequency to the facility.^34^ Community Drug Distribution Points (PODI), ART distribution points managed by PLHIV who are trained to provide ART refills, adherence support and follow-ups have also been rollout in the Democratic Republic of Congo.^35^

### Methodological Approach

**Figure 2:**
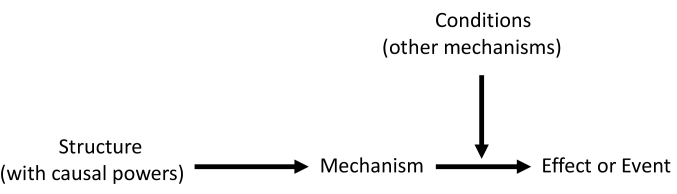
Conceptualising Causation in Critical Realism.^38^

Scientific research under critical realism strives to develop explanations for the way things work and how they are capable of doing so.^36^ Claims are made in critical realism by providing explanations on a set of events by hypothesising the existence of mechanisms – choices, reasoning and decisions of actors, responsible for generating the observed behavioural pattern. These mechanisms result from the interplay between actors and dynamic open systems – context or conditions (Figure 2).^37^

Central to critical realist methodology is, therefore, the identification and conceptualisation of mechanisms and the conditions or contexts hypothesised to trigger an event.^39^ To this end, we applied a critical realist informed approach to unearth how the resources and opportunities provided by DSDMs influence the perceptions and reasoning of men (generative mechanisms) towards adopting ART-friendly masculinities (effect) and consequently engagement in HIV care (event).

### Study Design

The case study design – extensive exploration of a single unit of study^40^ was adopted for this study because it is “compatible” with the critical realist research philosophy.^36^

### Study Setting

The Michaël Maphongwana clinic is a community health centre in Khayelitsha, Western Cape province, South Africa, which was identified by the Khayelitsha-Eastern Sub-Structure management in November 2015 to pilot 3 DSDMs; FACs, CACs, and QPUPs.

As at June 2017, the Michael Maphongwana clinic had 7091 patients retained in ART care. Of these, 2483 were retained in the adherence club care amounting to 35% of patients getting their care in either FACs or CACs. The facility has 110 adherence clubs; 50 CACs and 60 FACs. The QPUP model has 28 groups of patients with each group having an average of 45 patients. An estimated 1054 patients were retained in QPUP in June 2017.

The Michael Maphongwana facility was selected for this study because it is one of the facilities in the sub-structure with the highest HIV cohort and one of the few public health facilities implementing all 3 DSDMs. With most of the patients on ART at the facility being stable patients, the FAC reached maximum capacity as determined by the available resources. The facility consequently introduced the CACs and the QPUPs to further decentralise ART delivery and broaden patients’ preferences on the option of care to reduce clinic congestion.

## Methods

A qualitative critical realist approach informed by the critical realist paradigm was adopted. Qualitative realist research approaches are used to promote the description of information relevant to the underlying structures and mechanisms to explain an observation or behavioural pattern.^41^

### Participant Selection

The study population included all men using DSDMs in the Michael Maphongwana clinic. An average of 5 attends an adherence club and 10 per QPUP in the Michael Maphongwana clinic. DSDMs having 6 or more men were purposively identified for participation. The researcher attended the sessions of the identified models and with the help of the model facilitator, the study aims and the roles of the participants were explained to the men in the model. The group members were informed that if they consented, in their next visit, they would be having a focus group discussion (FGD) with some members potentially taking part in in-depth interviews (IDIs). The men who attended the sessions were gathered to take part in the FGDs.

**Figure 3:**
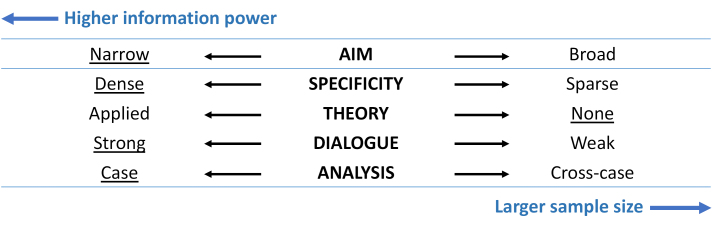
Sample Size Estimation Based on the Information Power Concept. The underlined elements are the defining characteristics of this study.

A selective purposive sampling approach – the identification of populations and settings prior to data collection^42^ was adopted to initiate the sampling process. The selective sampling was informed by the concept of *information power*, the more relevant information the sample holds, the lower number of participants is needed.^43^ Following the information power framework, the study was classified as having a high information power thus requiring a small sample size (Figure 3). Participants were recruited based on the potential information that they could provide toward the theory construction.^44^ Using this approach, 21 participants were recruited (Table).

**Table:** Characteristics of the Study Participants

	**QPUP**	**CAC**	**FAC**	**Total**
FGDs (2 per DSDM)	8	7	6	21
IDIs	5	5	5	15
Age				
25–35	5	3	1	9
36–45	2	4	4	10
46+	4	3	2	9
Marital status				
Married	4	3	3	10
Single	2	2	2	6
Divorced	3	2	1	6
In a relationship	2	3	3	8
Employment status				
Employed	9	9	7	25
Unemployed	2	1	2	5

Abbreviations: FAC, facility-based adherence club; CAC, community-based adherence clubs; QPUP, quick pharmacy pick-ups; FGDs, focus group discussions; DSDM, differentiated service delivery model; IDIs, in-depth interviews.

When the first sets of data were analysed, it was realised that more theoretical data were required to substantiate the emerging model. To this end, further 9 participants, 3 from each model were recruited before theoretical saturation was achieved – continued sampling and analysing data until no new data appear and all concepts in the theory are well-developed.^45^ The use of the information power framework is in line with the suggestion of Vasileiou et al^46^ that qualitative authors should consider a range of factors that influence sample size sufficiency and the specifics of a particular study.

### Data Collection

Six FGDs were conducted with 3 to 5 participants per group; 2 with participants using each DSDMs – FAC, CAC, and QPUP. FGDs were used to initiate group discussions on how these models reshape masculine identities and promote their engagement in HIV care. The FGDs also allowed the researcher to explore the beliefs and shared meanings of the men in DSDMs. Each FGD was conducted at the place where the model activities are held. For instance, facility boardroom for FACs or church hall for CACs and each FGD lasted between 45-60 minutes.

The moderator explored the shared practices and habits of the men using the models and their shared understandings associated with these practices.^47^ The questions were related to (1) Why men are reluctant to test for HIV and initiate treatment; (2) the preferred modalities of the DSDMs; (3) the importance of those modalities with regard to self-management of the disease; (4) How these modalities help them to regain their life; and (5) why it is important to remain in care and adhere to medication. The similarities and differences in opinion during the discussion sessions were noted and further explored in the IDIs.

IDIs were conducted with participants selected from FGDs corresponding to the different DSDMs, allowing the interviewer to uncover deeper understanding. IDIs helped explore the change of views, perspectives and behaviours of men using DSDMs. Because the IDIs were an extension of the FGDs, they only lasted between 10–20 minutes and were conducted in a private room secured for this purpose. Open-ended questions were used to initiate the flow of information and semi-structured in-depth questions were then used to explore various aspects of the phenomenon.

A further 9 IDIs were conducted after the initial data collection aimed at substantiating the data. These 3 questions guided the additional IDIs.

- In what ways have the FAC, CAC or QPUP encourage you to always come to the clinic and always take your medication?
- Can you explain how it has helped you to regain your life, respect and position in the community?
- How has it helped you to take care of your family?

FGDs and IDIs were conducted in isiXhosa as this was the preferred language of the participants. The first sets of data were collected between May and July 2019 and the second set was collected in November 2019. All FGDs and IDIs were recorded with permission from the participants. The audio-recordings were transcribed verbatim in isiXhosa and translated by the research assistant who is fluent in both English and isiXhosa. The transcripts were checked by the author for consistency and appropriateness by comparing with the questions on the guides.

### Data Analysis

Data obtained from FDGs and IDIs were first prepared into transcripts and fed into the Atlas.ti version 9 data management software. The goal of the data analysis was to identify theoretical constructs informed by critical realist concepts of mechanisms (reasoning individuals apply to available resources) and context (salient conditions that are likely to enable or constrains mechanisms).^48^ To this end, we adopted the data analysis approach proposed by Strauss and Corbin^49^ guided by retroductive thinking^50^ – a form of inference to identifying and verifying mechanisms that are theorised to generate the phenomena.^51^ Codes were generated as the first stage of reducing the data.^41^ The open coding followed a non-exclusive indexing approach to avoid selection bias at the early stages. An inductive axial coding technique was then used for identifying codes related to causal mechanisms and contextual elements. The process of abstraction was then applied to classify the initial codes obtained from the coding processes into conceptual categories,^47^ presented as thematic networks^52^ – web-like illustrations that summarize the main themes. The conceptual constructs obtained are considered as nuggets of information or building blocks required for theory construction (data synthesis).

### Data Synthesis

Data synthesis was guided by retroductive thinking and abductive theorising – identifying the likeliest possible explanation for the dataset.^53^ Through the process of retroduction, each mechanism (M) or bundle of mechanisms (M) was linked to the observation (O) of interest (M-O links).^54^ Retroduction was also applied to identify the necessary contextual (C) conditions modifying the causal mechanism to take effect.^53^ Because retroduction offers the possibility for multiple potential explanations,^36^ we had to adjudicate between rival or competing C-M-O links.

## Results

The results are presented in 2 sections: (1) Thematic constructs networks associated with mechanisms and context and (2) the overall explanatory model. The results illustrate the mechanism-related constructs explaining why and how DSDMs enhance men’s refashioning of ART-friendly identities and important context element (individual and health systems-related) that can facilitate or act as a barrier to the process of refashioning ART-friendly identities.

### Mechanisms

**Figure 4:**
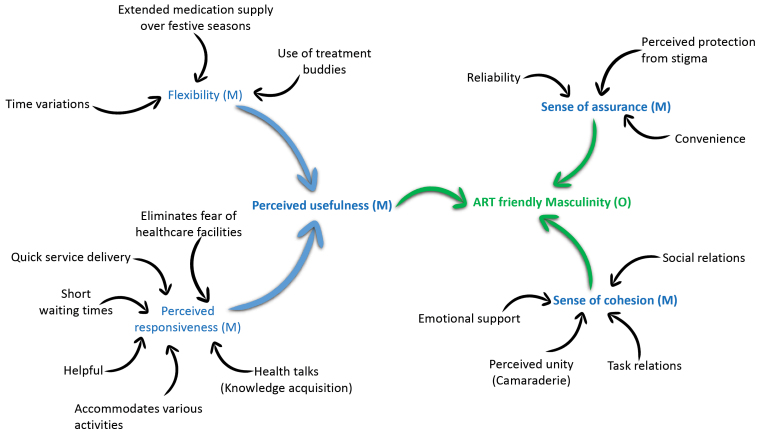
Coding Tree for Postulated Mechanisms. Abbreviation: ART, antiretroviral treatment.

Three bundles of mechanisms, perceived usefulness, sense of assurance and sense of cohesion, were identified to be responsible for promoting the process of reformulating ART-friendly masculinities by men using DSDMs. The process of identifying and postulating these bundles of mechanisms is illustrated in Figure 4.

#### Perceived Usefulness

Perceived usefulness relates to the extent to which an individual believes the model enhances their disease management and the ease with which men can fit their treatment into their lifestyle. Identified constructs related to perceived usefulness include perceived responsiveness and flexibility.

#### Perceived Responsiveness

Short waiting time is one of the most mentioned elements of responsiveness.

*“Yoh! That side [standard clinic care], you wait for ages. There are a lot of people and it takes longer for people to be served that is why I chose the club because here everything is quick. There is nothing that makes you wait for the whole day. Within an hour at the club, you are on your way to work”* (FAC; IDI; Male 28).

A participant indicated how the quick medication pick-up component of these models responded to their needs regarding carrying out various activities.

“*We can take our medication and go our separate ways as some are rushing to work, some are rushing home to take care of grandkids, some are rushing wherever*” (FAC; IDI; Male 60).

A QPUP participant explained how the model helps them to keep their jobs as it allows them to arrive at work on time. This aspect specifically enhances their ability to provide for their family thus reformulating their responsibility masculinity.

*“Men do not have time to wait like what is currently happening in the clinic. Because the main thing is to work and make a living”* (CAC; IDI; Male 56).

A 45 year-old-man using the CAC reflected that being in CACs also eliminates the notion of being in a healthcare facility, addressing one of the issues with hegemonic masculinities – fear of going to the clinic.

“*Here [CAC], you even forget that you are coming to the clinic. Yes, that is why I like it here…The process here is very smooth”* (CAC; FGD2; Male 45).

Another participant reported on the responsiveness of these models regarding relevant knowledge acquisition.

“*The club has given me more knowledge about different experiences as we walk in life with HIV. We shared experiences and challenges. I am close to 5 or 6 years in a club and it [FAC] has motivated me to remain in club care”* (FAC; FGD; Male 38).

#### Flexibility

Two participants also described the perceived flexibilityof the QPUP based on the operating hours, which enhanced their reconstruction of ART-friendly versions of masculinity.

*“I told her I wanted the QPUP one because I can come and collect after work. The time for QPUP is flexible for me”* (QPUP; IDI; Male 28).

“*Yes, I think it [the ART delivery model] might help us [men]. Some work nightshift and some work during the day. So, these models like QPUP might help us because it is flexible for them*” (QPUP; IDI; Male 37).

Some of the participants expressed that the flexibility of the DSDM is also related to the models allowing treatment buddies to collect their medication packages. This is another aspect that helps the men to perform other activities such as go to work.

“*Yes, my girlfriend normally collects for me especially when I know I will be late*” (QPUP; IDI; Male 37).

DSDMs also offer the flexibility of offering 4 month’s medication supply over the festive seasons to allow patients to travel while having a good supply of medication.

“*I have just spoken to my facilitator that I am thinking of going to the Eastern Cape to do some business there. So, I should have gone yesterday but had to wait for today so that I meet them in person. She has suggested that I get a transfer but in the Eastern Cape, access to ARVs is a challenge. So, she told me that we are getting 4 month’s supply right now [November], which I feel it is very good for me*” (CAC; IDI; Male 37).

#### Sense of Assurance

The mechanism of *sense of assurance* relates to men being convinced that DSDMs will fit in their lives by providing reliable, convenient and stigma-free services.

The reliability that these models are explained by a 50-year-old male in CAC.

*“I started at work and reported that I will be going to the clinic for a few minutes. Within no time I was back at work. I do not need to ask for off days*” (CAC; IDI; Male 50).

Participants in each of the models reported on the reliability concerning having their medication available when they arrived for refills.

*“Otherwise, every time I come here; I always get my mediation… I always arrive here, and my medication is there when it is time to get meds*” (CAC; IDI; Male 45).

“*Here [QPUP], you find all your medication ready to pick up”* (QPUP; IDI; Male 37).

A participant also suggested that men are easily retained in ART care through DSDM because they offer convenience to them.

*“Nothing can delay us here. Some are coming from work already and some are still going. So, it is very convenient for everyone. At the end of the day, we are all not delayed”* (CAC; IDI; Male 45).

Another participant spoke of the convenience that QPUP offers with regards to adapting their disease management into their lifestyles.

*“So, you can schedule your activities around. For example, when I am working, I am able to ask from work to knock off a bit early”* (QPUP; IDI; Male 28).

Stigma is a strong deterrent to the development of ART-friendly masculinities as it is tied to the reputational identity. Participants also highlighted that DSDM, especially the adherence clubs, incite perceptions of being stigma-free.

*“This place is not crowded [CAC]. No, people are walking around here and when we start our club at 7 am, you do not want to be always seen by people who start asking questions on what happens here. I take my treatment but when people start looking at you and asking questions it makes me sometimes feel uncomfortable*” (CAC; IDI Male 45).

*“I only come here when there is no one and get my medication and leave. It is always empty when I come here”* (QPUP; IDI; Male 37).

#### Sense of Cohesion

Group cohesiveness arises when bonds link members of a social group to one another and the group as a whole. Sense of cohesion is informed by 4 sub-constructs: social relations, task relations, perceived unity, and emotional support.

Social relations speak to the shared experiences, bond and support among patients in the models. It is mostly provided by FAC and CAC but not in the QPUP models. This is because the adherence club models provide an environment where the participants can meet and discuss.

*“If you are not feeling well, they can also advise on what to do since we are also on the same journey. Maybe I might have gone through the same thing. So, I will share my experiences*” (CAC; FGD; Male 45).

Emotional support relates to the extent to which a person feels they can cope based on their relations with others. Emotion support is enforced by the social support that some of the differentiated models provide. A FAC participant reported on the supportive role that he provided to another club member. Providing emotional support to others helps build respectable masculinities, which is associated with a man acting as an inspiration and adviser to others.

*“I have been a supportive friend to him, I hope. I have been encouraging him to take his medication as required”* (CAC; IDI; Male 45).

Perceived unity aligns with sharing a bond as group members. One of the participants suggested that the bond that they form transcends club activities and extends out of the club.

*“In terms of emotional support, we have friends and relations with other club members to chat even out of the club”* (FAC; FGD; Male 38).

Task relations speaks to working together toward achieving a common goal. A 35-year old man recounted how they support each other toward achieving the goal of medication adherence.

*“Yes, we encourage each other to take medication. It is not easy … but we decided to be there for each other*” (FAC; IDI; Male 35).

### Relevant Context Conditions

**Figure 5:**
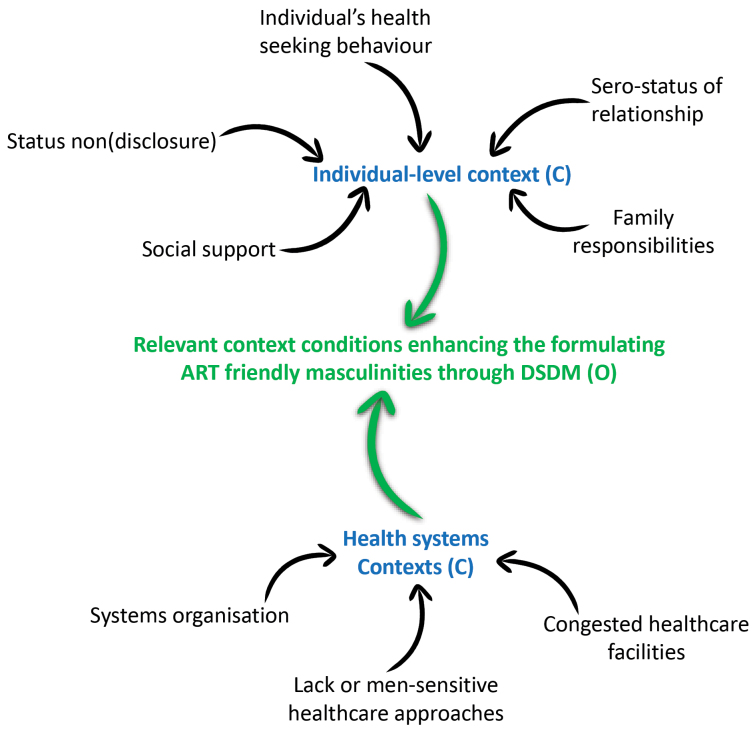
Coding Tree for the Context Elements. Abbreviations: ART, antiretroviral treatment; DSDM, differentiated service delivery model.

The context elements are presented under health systems (meso) and individual-related micro-context. Figure 5 illustrates the coding tree of the context elements.

#### Health Systems Context

Healthcare delivery approachwas identified as an important contextual element influencing the delivery of DSDM. One of the participants suggested that the current HIV service approaches adopted in the healthcare facilities should speak to the needs of men to capture their involvement.

*“I think there is a need to directly talk about what affects men directly in their own space. Men cannot just open up and talk in open space. There should be messages spoken directly to them and those messages should not be general”*(CAC; IDI; Male 39).

The congestion and long waiting times experienced at the main clinic HIV treatment scheme enforces the relevance of the DSDMs on enhancing masculinity refashioning.

*“Most of the times, things are very slow at the clinic. You wait and wait, and this makes most people panic because we were meant to be going to work*” (QPUP; FGD; Male 40).

The well-organised nature of the services delivered at the DSDM enhances the services provided at the facility, which facilitated participation.

“*On my appointment date, I go, and my pills are always there. Even when I come for blood and clinical visits, I always find my parcels well-prepared by the facilitator. Everything in the club is prepared. Even when it is time for clinical visits when we need to see the nurse, our folders will be ready*” (FAC; IDI; Male 45).

#### Individual-Level (Micro-)Context

Most of the individual-level contextual elements that influence how DSDM work are related to masculinities. These include non(disclosure), social support, sero-status of relationship, family responsibility and health-seeking behaviour.

Status (non)disclosure is identified in the literature as an important individual-level context. A 42-year-old man reported that when he disclosed his HIV status, it was easy for him to access HIV treatment services.

*“I am open to my family. Even when it is 8 and I am sleeping, you will hear, ‘here is your water and tablets*’” (FAC; FGD; Male 54).

According to 1 participant, men display worse HIV disclosure behaviours compared to women. Poor disclosure constitutes a pertinent contextual element to reframing masculinities as it reduces social support.

*“But what I have realised is that men are not the same as women. It is very difficult [for men] to even disclose to their partner while they are lying in bed and having sex. But a woman would tell you at the beginning of the relationship and tell you to choose if want to you stay or not. But for men, they hide their status”* (CAC; FGD; Male 40).

Another important contextual element is being in a sero-(dis)concordant relationship. When both partners are HIV positive, it is easy to support each other in aspects such as medication pick-up, reminders and encouragement. One participant expressed how the support he receives from wife facilitates his engagement in HIV services.

*“My wife encouraged me to test because I had TB. She also came to the clinic and she found out that she was positive”* (FAC; IDI; Male 30).

Social support from family after disclosure to a significant other promotes participation in HIV health services. One participant gave his personal experience:

*“My family knows that I take medication and they support me. Even late when it is time to take medication, they remind me to take my tablets… And no one has ever judged me about my status”* (QPUP; IDI; Male 40).

Another participant expressed that his drive to engage with HIV services relates to the responsibilities he has towards his family.

*“I want to live because I have a family. My family is my all. For instance, I have children that I need to support. So, tell me, if I am careless and not take my meds, what will happen to them?*”

How DSDM will enhance the reframing of ART-friendly masculinities depends on their health-seeking behaviour, which is tied to their masculinities. Participants suggested that men do not want to be associated with HIV as it affects their reputational masculinity.

*“They [men] do not want to be seen and associated with it [HIV]. Even for me, it was not easy to go and test. I went to [take an HIV] test because I was very sick*” (FAC; IDI; Male 30).

### Theoretical Model

**Figure 6:**
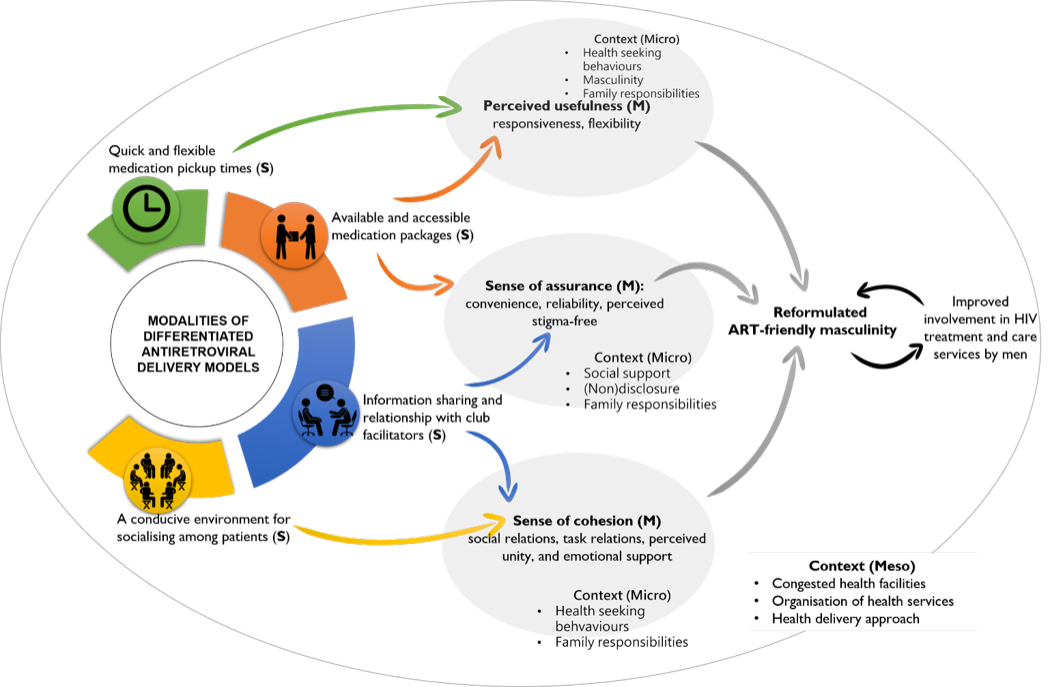
A Causal Model Illustrating the Generative Process of Reformulating ART-Friendly Masculinities. Abbreviation: ART, antiretroviral treatment.

Following the application of retroduction and abduction, a model describing how DSDM enforces the involvement of men in ART programmes through the refashioning of ART-friendly masculinities was configured (Figure 6).

## Discussion

The aim of the study was to tease out an explanation of how and why the modalities of DSDMs enhance the participation of men in HIV treatment and care services by modifying hemogenic masculinities to ART-friendly masculinities. Three bundles of mechanisms driving the adoption of ART-friendly masculinities by men using DSDMs were identified. (1) DSDMs provide a *sense of cohesion* (social support and feeling of connectedness), which enhances men’s reputational masculinity – having the know-how and being knowledgeable. (2) DSDMs provide a *sense of assurance* by providing reliable, convenient, stigma-free services, which makes men feel strong and resilient (respectability identity). (3) Through *perceived usefulness* (the extent to which an individual believes the model enhances their disease management), DSDMs enhance men’s ability to be economically productive and take care of their family (responsibility identity).

Perceived usefulness was identified as an important mechanism driving the refashioning of ART-friendly masculine identities. Perceived usefulness stems from the easy access and the short time spent to collect medication at DSDMs. Eshun-Wilson et al^55^ demonstrated that stable ART patients using DSDMs prefer infrequent clinic visits and the short time spent for ART pick-up. Important systems contextual elements associated with the timely supply of medication at the DSDMs are good organisation and availability of medication.^56^ This study unveils that the quick services provided by DSDMs offer men flexibility by accommodating them in various ways regarding their jobs and other activities. Receiving quick ART services at their convenience enhances men’s ability to work and earn, which restores their responsibility masculinities.^16^ Another study identified mechanisms such as perceived benefit, motivation and satisfaction to enhance medication adherence and retention in care of patients in DSDM.^57^ Facilitating quick access to medication should, therefore, be a priority for policy-makers and health practitioners.

The findings also identified ‘sense of cohesion’ as an important mechanism of men’s refashioning of ART-friendly masculinities. The social relations, task relations, perceived unity, and emotions shared by members of group-based DSDMs encourage and motivate men to participate readily and to remain in care and adhere to their medication. This explanation aligns with the reports of other authors suggesting that the camaraderie or cohesion among patients and the social support offered by the group members in adherence clubs encourage members to remain in care and adhere to their medication.^29,58^ Cohesion among group members and social support provided to each other speaks to the refashioning of the reputational masculinity, which relates to the notion of having the know-how. Having the shared responsibility to tackle HIV by sharing helpful experiences or giving advice to others in the models facilitate men’s refashioning of their responsible masculinities. Russell et al^59^ confirmed that by providing knowledge, support and encouragement, men living with HIV support each other in the initial process of refashioning their masculinities. The importance of group cohesion is accentuated in a study that found that adherence clubs that operate as *de facto* medication pickup without the gathering and interaction of patients left the patients dissatisfied and demotivated to engage in care.^60^ This finding can be indicative that adherence clubs group cohesion is pivotal in engaging men in HIV services. Policies and practices encouraging conducive conditions for group interactions such as providing convenient spaces where men can interaction can enhance patients’ engagement in ART care. Further cohesion such as mentoring, and role modelling can also be encouraged within the groups. In this way, men who show better ART-friendly masculinities could be encouraged to ‘mentor’ those who are struggling by actively pairing them.

A third mechanism hypothesised to driving the refashioning of ART-friendly masculinities of men using DSDMs is ‘sense of assurance.’ The relevant health talks and counselling sessions that men receive from the counsellors provide a sense of assurance to the men, which in turn motivates them to remain in care and adhere to their medication. This finding is congruent with that of Skovdal et al^61^ who explained that properly counselled men who have had the opportunity to reflect upon the impact of ART on their productivity and social value construct new and more ART-friendly versions of masculinity, which improves their engagement in ART services. Findings from a study conducted by Sileo et al^62^ confirmed that continuous reminder of the benefits of ART enforces men’s ability to fulfil the roles important to them, which further engages them in HIV services. Another study found that providing health talks and counselling to PLHIV using DSDMs increases their self-efficacy, one’s perception of their ability to accomplish a task,^56^ an important mechanism driving the formulation of ART-friendly masculinities. We recommend that health talk should continue to be provided to men using DSDMs and when selecting models for implementation, preference should be given to models that provided health talks.

Because the respectability masculinity (being strong, resilient and disease-free) is related to how the community views an individual, Russell^15^ explained that with regard ART, respectability in the wider community could be refashioned with men acting as an inspiration and advisers to others. With the knowledge and experiences accrued in the DSDMs, men can fulfil this role in the community. However, they would need to be willing to disclose their status not only to their family and friends but to the entire community. Regarding the reframing of reputational masculinity, the marks of honour and status among fellow men, DSDMs contribute little. This is because the messages that are provided and the behaviours that are encouraged by DSDMs such as no alcohol consumption and no sexual promiscuity are contrary to the behaviours (alcohol consumption and multiple sexual partners) that promote reputational masculinity, which is mostly constructed among peers. In fact, Skovdal et al^11^ confirmed that traits of reputational masculinity (being in control, highly sexual and economically productive) are in direct conflict with the “good patient” persona men are expected to adopt being HIV positive, which requires engaging in health-enabling behaviours such as attending regular sessions visits and refraining from alcohol and unprotected extra-marital sex. Consequently, DSDMs, especially those having health talks aspects should include life-course perspectives to address issues on respectability and reputational masculinities.

Based on the findings of this study, while DSDMs contribute substantially toward the reformulation of the responsibility masculinities, they contribute little in the reframing of reputational and respectability masculinities. Therefore, DSDMs provide a minimal contribution to gender transformation aimed to challenge and transform local beliefs and practices that undermine their health-seeking behaviours. To improve the efficiency of DSDMs, aspects that aim to challenge and transform local beliefs and practices deterrent to good health-seeking behaviours should be considered especially during the delivery of health talks and counselling.

It must be said, nevertheless, as indicated by the study findings that DSDMs do not only improve the engagement of men by modifying their masculinities but that there is direct generative causation between the intervention modalities and men’s engagement in HIV services. Mantell et al^63^ explored the facilitators and barriers to HIV-positive men’s participation in DSDMs and unearthed that the men found the DSDMs convenient, efficient, providing solidarity, and mutual psychosocial support. By providing convenient, efficient services that promote solidarity and mutual support, DSDMs improve self-efficacy, motivate and empower people using these models to remain in care and adhere to their medication.^31,57^ Nevertheless, to achieve better engagement of men in HIV services, DSDMs should include gender-transformative messages to facilitate men’s refashioning of ART-friendly masculinities.

### Study Limitations

The findings of this study were obtained from a single case thus limiting the possibility of generalising the findings to other populations other than the case-related population.

Conducting studies involving the exploration of theoretical constructs such as masculinity may be challenging especially among participants without extensive formal education. This challenge was addressed by (1) breaking down the concepts into simple notions reflecting the daily activities of the participants and (2) piloting the FGD and IDI guides; participants in the pilot were asked if they understood the questions asked and the subjects of discussion.

## Conclusion

Improving the participation of men in HIV treatment services requires addressing not only the structural barriers to access to HIV health services but would also require targeting and adapting interventions to meet the needs of HIV positive men. To improve male HIV service participation, health systems need to, apart from addressing the health systems barriers to access to care and treatment services, adopt ‘male-friendly’ approaches to address men’s specific HIV needs. The theoretical model developed in this study explicates how DSDMs contribute to the reformulation of ART-friendly masculinities, which in turn enhances their participation in HIV treatment and care services. With DSDMs already showing potential to attract and retain men in HIV treatment services, having an explicit and evaluable theory of what is working, for men and in what health systems context, can inform the reconceptualization of these models to enhance men’s refashioning of ART-friendly masculinities. Their effectiveness can be enhanced by adding aspects of gender-transformative motivational education to these models to foster the process of refashioning ART-friendly masculinities.

## Acknowledgements

The author acknowledges Ms. Sibusiso Ndlovu for her huge role in the data collection and preparation phases of the study. The author also acknowledges the contribution of Professor Stephanie Topp who provided valuable comments to a version of the manuscript. The author also acknowledges the vital role that the four anonymous reviews played in improving the quality of this article.

## Ethical issues

This study received ethics approval from the University of the Western Cape’s Biomedical Research Ethics Committee (BMREC): Reference – BM19/1/18. Research ethics was also obtained from the Western Cape Province’s Research Ethics Board: Reference – WC_201903-029. Permission to conduct the study was also sought from the Community Health Facility where the study was conducted.

Informed consents were obtained from all study participants before their participation. The purpose and objectives of the study were provided to each participant prior to their enrolment into the study. The informed consent also detailed the voluntary nature of the participation to the participants emphasising their right to withdraw at any phase of the research FGDs or IDIs. Confidentiality was ensured at all stages of the study that is why pseudonyms are used to en-sure confidentiality while reporting the findings.

## Competing interests

Author declares that he has no competing interests.

## Author’s contribution

FCM is the single author of the paper.

## Endnotes


[1] ‘Engagement in care,’ was considered to encompass ‘retention in care’ and ‘adherence.’^7^


## Key Messages

**Implications for policy makers**

- Policies that strengthen the existing antiretroviral supply chains are critical as timely dispensing to patients can contribute to sustaining and improving men’s participation in HIV services.
- Models that encourage the grouping and interaction of men living with HIV should be prioritised over models that do not.
- Adapting differentiated service delivery models (DSDMs) by adding gender-transformative educational components to enhance the process of refashioning men’s masculinities should be considered.

**Implications for public**

Functional and practical support such as picking-up medication for men living with HIV from their HIV treatment models can enhance their treatment adherence. Emotional and psychological support provided to men in their families and communities reassuring their worth can help them to reposition themselves in their families and society
